# Evaluating the effectiveness of automatic image captioning for web accessibility

**DOI:** 10.1007/s10209-022-00906-7

**Published:** 2022-08-22

**Authors:** Maurizio Leotta, Fabrizio Mori, Marina Ribaudo

**Affiliations:** DIBRIS - University of Genova, Genova, Italy

**Keywords:** Web accessibility, Alt-text, Automatic image captioning, User testing

## Abstract

The web has become a fundamental tool for carrying out many activities spanning from education to work and private life. For this reason, it must be accessible to every user regardless of any form of impairment or disability. Images on the web are a primary means for communicating information, and specific HTML elements were defined to enrich images with textual descriptions, which can be read aloud by screen readers or rendered by braille displays. A relevant problem is that adding a text describing each image published on a website is a demanding task requiring a non-negligible effort for web developers. Several tools based on machine learning have emerged, which can automatically return descriptions for the images. In this work, we evaluate the correctness of their outputs by comparing the generated descriptions with human-defined references. More specifically, we selected 60 images from Wikipedia and their corresponding descriptions as defined by Wikipedia contributors. We then generated the corresponding descriptions employing four state of the art tools (Azure Computer Vision Engine, Amazon Rekognition, Cloudsight, and Auto Alt-Text for Google Chrome) and asked 76 computer science students to blindly evaluate the perceived correctness of the descriptions without being aware of their source. The results show that the descriptions available in Wikipedia are still perceived as the best ones. However, some tools generate good results for specific categories of images, and they can represent proper candidates for the automated and massive addition of image descriptions to websites, helping to increase the accessibility level of the web drastically.

## Introduction

The web brought a remarkable development to the society, changing how we study and learn, work and do business, travel and enjoy. Social media, with around 3.6 billion users^1^ in 2020, have had a massive impact on how we communicate and share information, images, photos, ideas, and innovations. These changes affect all people, including those with different forms of disabilities. This was particularly evident during the COVID-19 pandemic lockdown when many activities and businesses were forced to move online.

The famous quote of 1997 by Tim Berners-Lee *“The power of the web is in its universality. Access by everyone regardless of disability is an essential aspect”* is even more relevant today: web accessibility is still of paramount importance since bad design choices or lazy use of existing authoring tools can create barriers that exclude people with disabilities from the current digital society.

Much work has been—and is still—done by the World Wide Web Consortium to make sure that everyone can participate on equal terms in the digital society. The Web Accessibility Initiative^2^ aims to spread the basic principles of accessibility and to promote best practices among developers, online content publishers, and social media contributors. Accessibility standards and guidelines have been published for websites and mobile apps developers, thanks to the Web Content Accessibility Guidelines [25] and the Mobile Web Initiative [23], respectively. If we focus on the accessibility of images, a good starting point is the tutorial [24] which explains how to provide appropriate text alternatives based on the purpose of the images themselves which can be informative, decorative, functional (e.g., the image of an icon), simple, complex (e.g., a bar chart, a line graph, a diagram), etc.

According to the Web Accessibility Initiative, *“Making a website accessible means allowing access to the information contained in the website also to people with different types of physical disabilities and those with limited hardware and software tools.”* Different forms of disabilities exist, from visual and auditory impairments to mobility and cognitive difficulties, and some people can have multiple concurrent disabilities. Moreover, almost everyone will experience temporary disability at one point in their lives, being in a situation of temporary fragility after an accident, during an illness, or simply because of poorer eyesight or other ailments due to aging.

In this paper, we will concentrate on visual impairments (for some recent data see for example [17]), a type of disability that needs special attention. Indeed, developers should guarantee access to online services and content to those people who have low vision or cannot see. Many assistive technologies exist to help them, e.g., screen readers, audio descriptions, magnification tools, refresh able braille displays, which can also be paired. For example, users with a combined hearing and vision loss can use a screen reader in conjunction with a braille display. Despite this, if the content itself is not accessible, reading can be difficult or impossible, even with the help of these assistive technologies.

People who cannot see cannot understand the message conveyed by an image, and an accurate description of its content is essential. To ensure access to an image for the blind, it is necessary to include an alternative text that can be read aloud by a screen reader or rendered by a braille display. The HTML markup language provides properties and elements to add such text, for example the alt and longdesc^3^ properties used in combination with the src element to include images in web pages, or the <figcaption> element used within the <figure> element. In this study, we will use the alt property and often adopt the term *alt-text* to denote image descriptions.

Describing online images and photos is time consuming, and, as a consequence, online content often fails to satisfy this essential accessibility requirement. If a picture is worth a thousand words, visually impaired users could be missing thousands of words of context.

Fortunately, in recent years, researchers in the AI community have developed algorithms and tools to automatically create natural language descriptions of images. This ability is essential for various tasks such as organizing extensive image collections or indexing and retrieving them in response to user queries. Many algorithms were proposed having these goals in mind. As a positive side effect, they can be used for accessibility purposes if the quality of the sentences they generate is good enough to be used as alt-text. It is also worth noting that the same alt-texts are indexable by search engines and provide quick summaries, which web crawlers use to understand the content of the images they crawl. These descriptions are also shown in place of the images when they are turned off, for example, in the case of mobile data roaming.

This paper evaluates the perceived quality of the descriptions generated by some tools developed for automatic image captioning. We limited the analysis to web images and collected a set of pictures from Wikipedia; their alternative texts were considered human-authored and formed the set of ground truth captions, e.g., the ideal reference of our experiment to compare the results provided by the various tools. We then formulated and answered the following Research Questions:

- *RQ1* Is there any difference in the perceived correctness among the descriptions generated by the considered tools?
- *RQ2* Is there any difference in the perceived correctness between the ground truth descriptions provided by humans and those provided by the tools?

To answer these questions, for each image, we queried the considered tools. We then evaluated the perceived correctness of their results thanks to a survey proposed to computer science university students attending Web and Mobile development courses (where accessibility is an important aspect).

This paper is organized as follows. Section 2 describes some related work and Sect. 3 introduces the tools chosen for the experiment. The survey administered to the students is presented in Sect. 4. The results are discussed in Sect. 5. Finally, Sect. 6 concludes this study suggesting possible future directions.

## Related work

Generating high-quality descriptions from images is a challenging task that requires interdisciplinary competencies at the intersection of computer vision, natural language processing, and machine learning. Details on different approaches can be found, for example, in [2, 9, 18, 21] where the authors present different algorithms, data sets, and evaluation metrics which have been proposed to generate image descriptions and to assess their quality with different levels of confidence.

AI researchers have developed algorithms that provide excellent results in the field of image classification, but automatically extracting a fluent description from a picture is much more complex. This task is not limited to object detection in a scene but involves recognizing faces or facial expressions, landmarks or specific points of interest, interpersonal relations, etc. To achieve this goal, it requires the use of sophisticated natural language generation techniques. Moreover, the evaluation of the quality of the results often requires a human intervention which is costly and does not always scale.

Data sets are needed for training, validating, and testing AI algorithms, and researchers have created many of them. We recall here the Microsoft COCO (Common Object in Context) data set [11] which collects *non-iconic* images, e.g., images containing objects in their natural context. This data set was indeed designed for the detection and segmentation of objects in a scene. From this collection, 91 categories were extracted (for example, person, bicycle, bus, dog), and five captions were associated with each image to generate new descriptions of new images. As the authors argue, the process adopted to build such a data set required a lot of work, which amounts to over 70,000 working hours of workers employed through the Amazon Mechanical Turk^4^ crowdsourcing marketplace.

Another data set, called Conceptual Captions, is presented in [20]. This data set was built by harvesting the web looking for a wide variety of $\langle image,caption \rangle$ pairs, including not only natural images (like in Microsoft COCO) but also other categories like products, cartoons, drawings, etc. The original alt-texts gathered from the web were transformed to obtain *conceptual* captions. This was achieved by removing proper nouns, dates, locations, etc., from the original captions or by replacing them, when possible, with the corresponding hyperonym, i.e., a word whose meaning includes the meaning of a more specific word. For example, “Lady Gaga” can be replaced by “singer”, “Tom Hanks” can be replaced by “actor”. This data set, which is much larger than other popular data sets, provides clean captions with fewer details but is still informative for training image captioning models.

Finally, we also mention the VizWiz data set^5^ which, differently from previous examples, is populated by people who are visually impaired. The pictures in this data set have a lower quality with respect to those chosen by sighted users and (optionally) can have an associated recorded question about the picture itself. The ultimate goal of this collection is to increase the awareness about the technological needs of people who are blind and to provide new opportunities for researchers to develop assistive technologies that eliminate their accessibility barriers.

We conclude the first part of this section citing two viewpoints. In a comment by Chiarella et al. [4] that appeared on Nature Communications, the authors observe that scientists increasingly post natural sciences images and photos on social media, but this content might be inaccessible to those with visual impairments when the alt-text is missing. They suggest that actions should be taken to guarantee access to these images and other multimedia objects to maximize and broaden education and research experiences.

Morris, in a recent article published in the Communication of ACM [14], poses some ethical considerations touching, among others, the problem of errors in AI algorithms. Many people with disabilities need to trust and rely on the output of an AI system without the ability to verify the output itself. Still errors may occur, even though sometimes popular press and advertising material wrongly states that these translation systems have reached “human parity”. The author concludes by saying that *“Educating our next generation of innovators is of paramount importance [...] As technologists, it is our responsibility to proactively address these issues to ensure people with disabilities are not left behind by the AI revolution.”* We agree and believe that education on accessibility is paramount for current and future developers to let them understand the social value of appropriate image descriptions and get into the habit of labeling them.

### Evaluating alternative texts

We introduce in what follows some works closer to our study since they specifically describe experiments related to the evaluation of image descriptions to be used as alt-texts.

Automated tests on web pages can be performed with validation tools to check whether they are accessible. These tools help to assess a minimal accessibility level, which is better than nothing. However, experts’ judgments is always required to capture subtler accessibility issues, as discussed for example in [22] or in a more recent study [3] where four commercial accessibility monitoring systems are compared.

In the case of images, the validation tools usually check whether alternative text is present or not. Unfortunately, the mere presence of such text does not guarantee accurate descriptions. The alternative text should indeed serve the same purpose as the non-text content: it should be *descriptive* and provide enough information without being too long. It should describe the content of an image without dwelling on visual details.

To make some examples, a common mistake is to use alt=“company logo” or even worse alt=“logo.png” to describe the logo of a company. These *undescriptive* captions provide little information when accessed with a screen reader and thus constitute a potential accessibility barrier. Other wrong alt-texts examples found in major websites are alt=“Image”, alt=“No photo description available”, or alt=“Insert alternative text here”, which can be automatically added by authoring software. Another common mistake is to include the two words “image of” in the alt-text since screen readers are already programmed to say aloud “image” when they encounter images on a page.

A discussion on descriptive vs undescriptive alt-texts can be found in [16] where the authors propose two approaches to automatically detect undescriptive alt-texts in web pages using pattern recognition algorithms. To get the data used for the classification, they analyzed the home pages of more than 400 Norwegian municipalities. By manually classifying the collected alt-texts either as descriptive or undescriptive, they found that 80% of the alternative texts in their data set were undescriptive, thus failing to correctly describe the corresponding content.

On the same line is the work in [1], where the authors analyze a set of images collected on some university websites and compare the results of human evaluation vs automatic evaluation done with the well-known AChecker validator^6^. In the university context, it is essential to carefully describe the complex images used for education purposes (for example, bar or pie charts, diagrams, scientific models of atoms or molecules, maps) so that blind students can access the same content of their sighted peers. Educators should be aware that the lack of these descriptions may constitute an accessibility barrier for students with disabilities. Moreover, for such complex images, assessing the quality of the corresponding descriptive text still requires a human evaluation.

The work in [13] compares two annotation methods for employing novice web workers to manually author descriptions for images in the STEM^7^ category, making them accessible to individuals with visual and print-reading disabilities. The first method introduced accessibility guidelines to the workers and let them free to construct image descriptions in an empty text box. The second method was more structured: templates were provided to the web workers to get the proper information. The captions generated with the two approaches are compared in terms of word counts, the inclusion of specific terms or categories, inclusion of units and data trends (when applicable), presence of syntactic errors, etc. The results show that guidelines are not sufficient for novice web workers to produce quality image descriptions, and it is better to generate such descriptions using templates. Moreover, the workers themselves preferred the use of templates and found the task easier.

The following two papers report about two different experiments done for the social media platform Twitter. The first [19] presents a browser extension, Twitter A11y^8^, which dynamically adds the alt-text to the images posted by the users. The generation of the alt-text is performed server-side using different methods, returning a result early if one is successful. The pipeline consists of optical character recognition, scene recognition, reverse image search, plus two additional methods specific to Twitter. If none of the prior methods produce a satisfactory alt-text, the extension asks a crowd worker on the Amazon Mechanical Turk to describe the image according to a set of guidelines.

The authors present the results of their experiments designed to measure the quality of the captions returned by the different methods and users satisfaction. They show that with Twitter A11y blind users were able to follow many more images. However, they observe that work still needs to be done to make content accessible on Twitter. There is also a need to educate users to describe their images since most of the photos lack an alt-text or have the default one, thus resulting inaccessible.

The second experiment on Twitter is described in [7] where the authors present a Conversational Assistant workflow that uses TweetTalk, a scalable conversational platform between visually impaired users and human assistants to find out about visual content. Analyses of the conversations collected from TweetTalk helped defining canonical questions such as “Where is this picture taken?” or “What action is happening in the image?” These questions might be useful for human captioners to describe the most relevant concepts visually impaired users need to better understand a scene. Some questions covered subjective issues, for example, “What emotion is evoked by the scene, or by the people in it?” Detecting emotions is currently an unsolved problem in AI.

The authors of [28] describe their Automatic Alt-Text system that applies computer vision technology to identify faces, objects, and themes from photos. The main goal is to present a useful, fast, free alt-text generation system for blind users of Facebook to enhance their online experience. The alt-text is constructed in the form of “Image may contain...”, followed by a list of objects recognized by the computer vision engine. The primary design decisions include the selection of object tags, the structure of information, and the integration of machine-generated descriptions with the existing Facebook photo experience. Again, selecting the right tags is not an easy task, and the authors ended up with a list of 97 concepts that provide different sets of information about the image, including people, objects (e.g., car, building, tree, cloud, food), settings (e.g., inside a restaurant, outdoor, nature), and other image properties.

Good feedback was provided during (1) lab interview sessions with few blind users and (2) a large-scale experiment with thousands of visually impaired Facebook users, split into test and control groups. Users in the test group used the automatic alt-text system. Those in the control group did not, and, as expected, the former had an easier time understanding the content of photos. However, several design challenges also emerged, the major one related to the quality of the tags. It is indeed possible to get more tags with less accuracy, but would blind users still trust the system in this case?

On the same line is the work presented in [12], where blind users evaluate the captions of Twitter images, and the results show that blind and visually impaired people trust incorrect AI-generated captions and fill in details to reconcile discrepancies rather than suspecting the captions may be wrong. Another interesting point discussed in this work is related to the framing of the captions, considering the effect of positive vs negative framing. Results show that negatively framed captions encourage more distrust on low confidence captions. Machine-generated captions can contain errors, and sometimes the algorithms can hallucinate objects [20]. While sighted users can easily ignore or correct the wrong captions, blind users cannot do the same and incorrect captions can lead to misleading messages.

We could not find papers that compare well-known commercial tools for the generation of image descriptions to be used as alternative texts. A research similar to ours, but focused on tag-based descriptions, is that of researchers at Perficient Digital Company, whose goal was to discover the best image recognition engine [6]. They looked at Microsoft Azure Computer Vision (Sect. 3.1), Amazon Rekognition (Sect. 3.2), Google Vision^9^, and IBM Watson^10^.

For their study, the authors selected 2,000 images in the four categories charts, landscapes, people, and products; three users tagged them manually. Then they evaluated the accuracy of the tags returned by the recognition engines and how well the results matched the human expectations.

For the accuracy, the results show that a tag could be judged to be accurate, even if it was one that a human would not have chosen in describing the image. For example, a picture of an outdoor scene might get tagged by the engine as “panorama”, and be perfectly accurate, but still not be one of the tags a user would think of to describe the image.

For the matching with human expectations, for each image, the manual tags and the top five highest-confidence tags from each engine were presented, without revealing the source. Users had to select and rank the top five tags that they felt best describing the images. Results show that the tags written by humans score far higher than any of the engines. This is to be expected, as there is a clear difference between a tag being accurate and a tag being what a human would use for describing something. Among the engines, the winner was Google Vision, of course after human captioners.

Before ending this section, it is important to keep in mind that captions of different types exist. Alt-text can be written as a list of *tags* or *keywords* describing the objects detected in the image; it can be a *conceptual description*, e.g., a fluent sentence in which more generic words replace specific data such as proper names; at the opposite side, it can contain *details of places* or *individuals*, for example in the case of celebrities, political figures, scientists, etc.

## Tools for the automatic generation of image descriptions

This section briefly introduces the four tools selected for the experiment; we chose them since they have many online reviews and seem to be among the most relevant for generating image descriptions. Moreover, some of them are proposed by big players like Microsoft and Amazon. They produce different results, from sequences of tags to structured sentences. With this experiment, we could assess the perceived correctness of their outputs when used for alt-texts.

In the remainder of this section, as a reference example, we will use a Wikipedia image^11^ showing four small quantities of different kinds of sugars characterized by different colors and having similar sizes. In particular, the image shows clockwise from top-left: white refined, unrefined, unprocessed cane, and brown sugar.

### Azure Computer Vision Engine

Microsoft Azure^12^ is a cloud platform that provides services for software development. The Azure Computer Vision Engine^13^ (Azure CVE for short) is one of such services which grants access to advanced AI algorithms focused on image processing. It is part of the Azure Cognitive Services^14^, a group of services that allow developers to easily add cognitive features into their applications without having AI or data science skills.

One of the most appreciated features of Azure CVE is the facial recognition which provides the ability to recognize famous people around the world. According to Microsoft blog^15^, *“Microsoft researchers have built an artificial intelligence system that can generate captions for images that are, in many cases, more accurate than the descriptions people write. The breakthrough in a benchmark challenge is a milestone in Microsoft’s push to make its products and services inclusive and accessible to all users.”*

A web developer willing to try Azure CVE can call a REST API which is available online^16^. By uploading an image as input, the AI algorithms process it and return a JSON file with the answer, e.g., a description composed of tags and complete sentences, with different confidence levels (see a portion in Table 1).

**Table 1:** Portion of the JSON file returned by Azure CVE

Tags	[“bear”,“teddy”,“indoor”,“stuffed”,“brown”, “sitting”,“close”,“food”,“cake”,“holding”, “table”,“laying”,“plate”]
Captions	[{“text”:“a close up of a teddy bear”, “confidence”:0.7086517}]
Format	“Jpeg”

The resulting text for the reference image is “a close up of a teddy bear” with a confidence level equal to 0.709 and it is clearly a wrong description, an example of object hallucination (the teddy bear).

### Amazon Rekognition

Amazon Rekognition^17^ (Amazon Rek for short) is an image analysis service available in the Amazon AI suite^18^. Like Microsoft Azure, also Amazon offers different AI algorithms that can be easily integrated into users applications: advanced test analytic, automated code reviews, chatbots are only just some of the many available services.

This tool employs a deep learning technology that requires no AI expertise to add labels to images and videos. The service allows to identify objects, people, text, scenes, and activities in images and videos, as well as, to detect any inappropriate content.

Differently from the Microsoft engine, the Amazon Rek service does not describe the content of an image, but it returns a list of tags describing the objects detected within the image. To access this vision engine, we used a third-party service^19^ which offers REST APIs. For the sugar test image, the returned tags are the words “sugar, food”.

### Cloudsight

Cloudsight^20^ is a company specialized in image captioning and understanding. Their on-device computer vision model can be used directly on users devices: it uses the device’s camera to photograph objects and identify them aloud for users with low vision.

This solution, announced in 2019 [5], drastically decreased the execution time for objects recognition. Indeed, according to their announcement, the algorithm can describe a picture in less than 250ms and: *“The process happens so fast on-device that the users may not even have to take a photo. Real-time streaming essentially means you can scan your phone around, and whenever you stop on an object and your phone focuses, the technology will immediately recognize the object.”*

The description of the sugar reference image of obtained with the Cloudsight algorithm is “brown powder on white ceramic plate”. Again, a hallucinated object (the white ceramic plate) is detected in the image.

### Auto Alt-Text for Google Chrome

Auto Alt-Text for Google Chrome^21^ (Auto Alt-Text for short) is a browser extension that can generate on-the-fly descriptive captions for pictures. By installing this browser extension, a screen reader can read aloud the captions of the images in the currently loaded web page, if available. When a caption is missing, it can be generated thanks to an AI algorithm transparently called by the browser.

Users can right-click on any foreground image element to use this extension, select “Get Image Info” from the drop-down menu, and get the caption. The caption for the sugar reference image is “a close up of a cake on a plate” that, again, hallucinates objects (cake and plate) not present in the image.

### Summary

Table 2 summarizes the descriptions obtained for the sugar reference image. Since this image seems confusing some algorithms, we also consider another simple image from the Wikipedia milk page^22^, a glass of milk on a uniform blue background, to highlight the differences in the results (see Table 3).

**Table 2:** Captions for the image showing different kinds of sugars

Azure CVE	a close up of a teddy bear
Amazon Rek	sugar, food
Cloudsight	brown powder on white ceramic plate
Auto Alt-Text	a close up of a cake on a plate

**Table 3:** Captions for the image with a glass of milk on a uniform blue background

Azure CVE	a glass of milk next to a cup of water
Amazon Rek	beverage, milk
Cloudsight	white liquid in clear drinking glass
Auto Alt-Text	a cup of coffee sitting next to a plate of food

Notice that even for these two simple images, with only one main object in the foreground and nothing in the background, some descriptions contain errors that sighted users can easily detect but might constitute a problem for users with visual impairments.

**Table 4:** Overview of the experiment

Goal	Analyze and compare the image textual descriptions generated by the vision engines described in Sect. 3 to understand if there are differences in terms of their perceived correctness and analyze the differences with respect to human-authored descriptions.
Research questions	*RQ1* Is there any difference in the perceived correctness among the descriptions generated by the considered tools?
	*RQ2* Is there any difference in the perceived correctness between the ground truth descriptions provided by humans and those provided by the tools?
Context	Objects: descriptions of 60 images selected from Wikipedia covering the three categories Human, Landmark, and General.
	Subjects: 76 students in computer science.
Null hypothesis	No effect on correctness (measured with a 5-point Likert scale).
Treatments	Five: Wikipedia (manual), Azure Computer Vision Engine, Amazon Rekognition, Cloudsight, and Auto Alt-Text for Google Chrome.
Dependent var	Perceived Correctness of the description with respect to the corresponding image.

## Experiment

This section reports the experiment in a structured way, following the guidelines by Wohlin et al. [27].

The *goal* of the study is analyzing and comparing the quality of the descriptions generated by the tools described in Sect. 3*with the purpose* of evaluating possible benefits from adopting them for automatic image captioning. Results of this study can be interpreted from multiple *perspectives*: (1) researchers, interested to empirically assess the quality of the descriptions generated by state of the art AI solutions; (2) practitioners, willing to understand if image descriptions can be automatically generated and thus adopted in their applications. The *context* of this study consists of academic students as *participants* providing a score to the textual descriptions of images (the *objects*) by means of online questionnaires. Since many different descriptions can be considered correct for a single image, we believe that relying on humans for evaluating the effectiveness of the selected tools is a fundamental choice because it allows to understand if the generated descriptions are perceived of “good quality” or “correct” also by humans. Moreover, such tools have been developed to provide image descriptions to humans (e.g., visually impaired users), so the evaluation provided by humans is valuable (and fundamental). Table 4 summarizes the main elements of the experiment.

### Images and textual descriptions (Objects)

The experiment took place in the last quarter of 2020 when we selected 60 images from Wikipedia, considering three categories of images: *Human*, *Landmark* and *General*. The Human category was further split into three sub-categories: *Paintings of famous people* (Famous Paintings), *Famous people of the 20th century* (Famous 1900), and *Famous people of the 21st century* (Famous 2000). This choice helped us to evaluate the different tools against different categories of images frequently present in the web.

Famous Paintings and Famous 1900 pictures were collected from the first section of the Vital Articles Wikipedia page^23^, which is a page containing a list of subjects (and related links) for which the English version of Wikipedia has the most important articles. Also Landmark and General photos were collected from the Vital Articles page, in the Geography and Everyday life sections, respectively. Finally, Famous 2000 images were chosen from the first five articles of the Forbes Celebrity 100 ranking, available in the corresponding Wikipedia section^24^.

The images used in the experiment are those reachable by clicking on the small image in the top right part of each Wikipedia article (i.e., the main representative image for such Wikipedia entry). For each image we associated a set of five textual descriptions: the first is the alternative text written by the human contributors to the online encyclopedia^25^, while the other four are the descriptions generated by the tools described in Sect. 3.

The complete replication package containing the 60 images and the corresponding descriptions is available at:

https://sepl.dibris.unige.it/2021-AltText.php

### Questionnaires

From a pilot experiment conducted with two students, we noticed that the evaluation of the five descriptions of a single image required, on average, about 60-90 seconds. Thus, to limit the effort of the participants, we decided to split the 60 images in two groups, and we prepared two questionnaires—to be completed in about 30-45 minutes—containing 30 images each.

The questionnaires were implemented using the Feedback module of the educational platform based on Moodle^26^, hosted by our university. To avoid habit in the evaluation, the five descriptions were presented in random positions so that respondents could not associate a description to a particular tool by considering their repetitive orderings. Moreover, respondents were not aware of the origin of the descriptions.

For each image, we asked: *“Evaluate each of the following five descriptions for the picture above. In your opinion, are they good descriptions of the picture? (evaluate aspects like the correctness and the precision of the description).”*

The possible answers used a standard 5-point Likert scale with values: Strongly disagree (1), Disagree (2), Neither agree nor disagree (3), Agree (4), and Strongly agree (5).

### Participants (Subjects)

We advertised the questionnaires among students attending two courses of the 1st semester of the academic year 2020-21: Web development offered in the 3rd and last year of a Bachelor degree in Computer Science, and Mobile development offered in the 1st year of a Master degree in Computer Science. These two courses should introduce the issue of accessibility so that future developers are at least aware of the problems some categories of users might face while surfing the web or using mobile apps.

We used the Moodle forum of each course, asking interested students to participate and providing the links to the questionnaires. They were invited to answer questionnaire number 1 or number 2 depending on their matricula number, odd or even, respectively. We did not promise any reward to respondents.

In the instructions accompanying the announcement, we suggested participants, if necessary, to use a dictionary/translator for the descriptions to avoid language troubles such as a word in the description whose meaning was not clear/known (e.g., a peculiar word describing a specific object). The English skills of the participants are actually good being on average between level B2 and C1 of CEFR^27^. Many of them attend(ed) university courses in English, and they have also oral exams in that language. In our opinion, their skills are more than adequate to evaluate quite simple descriptions like those generated by the considered tools and proposed in the experiment. Indeed, note that the median length of the descriptions (including stop-words) varies from 4 words for Wikipedia (manual) to 9 for Auto Alt-Text for Google Chrome; only about 10% of the descriptions is composed by ten or more words; the maximum description length is 18 words.

We also assigned a specific interpretation for each value of the Likert scale, suggesting students to select one of the following options:

1. Strongly disagree: if you think this is a *totally wrong* description;
2. Disagree: if you think this is a *wrong* description, but with some objects or aspects rightly recognized;
3. Neither agree nor disagree: if you think this is a quite *vague* description;
4. Agree: if you think this is a *correct* description (but not 100% precise of what you can see in the image);
5. Strongly agree: if you think this is a *precise* description.

We had 76 questionnaires completed, for a total of 2280 evaluations for each kind of description and 11400 evaluations overall (5 for each image); 50 out of 76 respondents were bachelor students, the others, e.g., 26 out of 76, were master students.

**Fig. 1:**
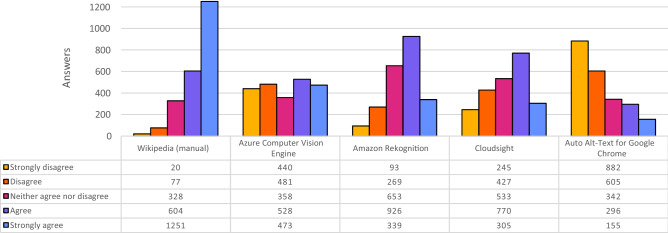
Overall results of the experiment, partitioned for each tool. The colors for the bars are chosen from the IBM design library “color blind safe” color palette [10]

### Variables and hypotheses formulation

Our experiment has one *independent variable* (also called “main factor” or *treatment*) with five possible levels:

1. Wikipedia (manual);
2. Azure Computer Vision Engine;
3. Amazon Rekognition;
4. Cloudsight;
5. Auto Alt-Text for Google Chrome.

The experiment has one *dependent variable*, on which treatments are compared: the perceived *Correctness* of the description with respect to the corresponding image. We can state the null hypotheses in this schematic way:

$$\begin{aligned} H_{0}: {Correctness} \text { (treat-A) } = {Correctness}\text { (treat-B) } \end{aligned}$$

where treat-A and treat-B are all the 20 different combinations of the five levels treatments: clearly we can exclude the five cases where the two treatments (A and B) are actually the same since they are of no interest. Since we could not find any previous empirical evidence that points out a clear advantage of one treatment vs the others, we formulated $H_{0}$ as non-directional hypotheses. The objective of a statistical analysis is to reject the null hypotheses above, so accepting the corresponding alternative ones, $H_{a}$:

$$\begin{aligned} H_{a}: {Correctness}\text { (treat-A) }\ne {Correctness} \text { (treat-B)} \end{aligned}$$

### Analysis procedure

After computing descriptive statistics, we used a paired Wilcoxon test [26] to compare the effects of the treatments on each subject. In all the performed statistical tests, we decided, as it is customary, to accept a probability of 5% of committing Type-I-error ($\alpha$) [27], i.e., rejecting the null hypothesis when it is actually true. While the statistical tests allow checking the presence of significant differences, they do not provide any information about the magnitude of such differences. Therefore, we used the nonparametric Cliff’s delta (|*d*|) effect size [8]. The effect size is considered small for $0.148 \le |d| < 0.33$, medium for $0.33 \le |d| < 0.474$ and large for $|d| \ge 0.474$.

## Results

In this section, first, we provide an overview of the results by analyzing some charts summarizing the answers’ distributions for each category of images. In this way, it is possible to understand how the respondents rated the descriptions generated by each tool. Then, in the second part of the section, we compare the results obtained by the various tools (and Wikipedia) using statistical tests to understand if the differences among the rates assigned to their descriptions (if any) are statistically significant or not.

### General overview

Figure 1 shows five histograms representing the distributions of the answers provided by the 76 participants for each kind of description. Each histogram summarizes 2280 answers since each student evaluated 30 images over a sample of 60 (30 × 76 = 2280). In addition to the histograms, Table 5 reports the summary statistics of the answers provided.

**Table 5:** Statistics of all answers, partitioned for each tool

	Wikipedia	Azure	Amazon	Cloudsight	Auto
		CVE	Rek		Alt-Text
Mean	4.31	3.05	3.50	3.20	2.23
Median	5	3	4	3	2
StDev	0.90	1.43	1.01	1.20	1.27

**Table 6:** Descriptions and answers for the image of the Manhattan skyline

	Strongly disagree	Disagree	Neither agree nor disagree	Agree	Strongly agree
*Wikipedia (manual)*					
Lower Manhattan skyline - June 2017	0	0	6	7	25
*Azure Computer Vision Engine*					
a large body of water with a city in the background	5	8	15	9	1
*Amazon Rekognition*					
high rise, city, urban, building, architecture	0	4	14	18	2
*Cloudsight*					
city skyline under blue sky during daytime	0	0	6	25	7
*Auto Alt-Text for Google Chrome*					
a large body of water with a clock tower on top of it	19	14	4	1	0

From the histograms and the values reported in the table it is evident that the human-authored captions received by far the highest scores. On a total of 2280 evaluations, only in 20 cases (<1%) the participants strongly disagreed with the proposed descriptions. On the contrary, in more than half of the cases, the participants evaluated the descriptions with the maximum score. This is a shred of clear evidence that the alt-texts written by Wikipedia contributors have a high perceived quality.

The results of the four AI algorithms are quite different. Indeed, Amazon Rek and Cloudsight generated the best descriptions according to the experiment participants. This is evident by looking at their histograms that are skewed to the right (i.e., toward positive evaluations on the 5-point Likert scale). It is interesting to note that, differently from Wikipedia, in the case of Amazon Rek and Cloudsight the most popular evaluation is Agree (respectively 40.6% and 33.8% of the cases), while the top evaluation (i.e., Strongly Agree) has been selected only in 14.9% and 13.5% of the cases, respectively. After these two positions, the third in terms of evaluation mean is Azure CVE: even if the average score is only slightly lower than that of Cloudsight, the distribution of the answers is very different. Indeed, Azure CVE shows a quite uniform distribution. As we will see in the following, this tool was generally quite accurate on specific categories of images but performed quite badly on others. Finally, participants evaluated the descriptions generated by Auto Alt-Text with the lowest grades. In this case, the distribution is clearly skewed to the left (i.e., toward negative evaluations). In 38.7% and 26.5% of the cases, participants strongly disagreed or disagreed with the automatic captions respectively.

To get a more detailed view of the evaluations, we consider as an example an image representing the Manhattan skyline^28^. Table 6 reports for each description the number of answers on the Likert scale. Alternative text should describe the information contained in a picture and not the picture itself, and the results show that the precision of the description is an important factor during the evaluation process. Almost all descriptions provide a correct representation of the image (except for an incorrect part in the description generated by Auto Alt-Text), and the evaluations reflect their accuracy.

We observed very similar results considering: (1) the two questionnaires Q1 and Q2 (see Table 7), and (2) the two groups of students from the Mobile and Web development courses (see Table 8). For this reason, in the following analyses we will not partition the results on these two categories.

**Table 7:** Statistics of all answers, partitioned for each tool and for each questionnaire

	Wikipedia		Azure CVE		Amazon Rek		Cloudsight		Auto Alt-Text	
Quest.	Q1	Q2	Q1	Q2	Q1	Q2	Q1	Q2	Q1	Q2
Mean	4.39	4.23	3.09	3.01	3.52	3.49	3.25	3.16	2.10	2.35
Median	5	5	3	3	4	4	3	3	2	2
StDev	0.85	0.95	1.47	1.39	0.99	1.04	1.17	1.23	1.17	1.35

Each questionnaire contains 30 images and 150 image descriptions; 38 participants answered to each questionnaire

**Table 8:** Statistics of all answers, partitioned for each tool and for each course

	Wikipedia		Azure CVE		Amazon Rek		Cloudsight		Auto Alt-Text	
Course	MD	WD	MD	WD	MD	WD	MD	WD	MD	WD
Mean	4.19	4.37	3.08	3.03	3.93	3.28	3.33	3.14	2.34	2.17
Median	4	5	3	3	4	3	4	3	2	2
StDev	0.89	0.90	1.40	1.45	0.85	1.21	1.15	1.23	1.26	1.27

26 participants are from the Mobile development (MD) course and 50 are from the Web development (WD) course

### Wikipedia

**Fig. 2:**
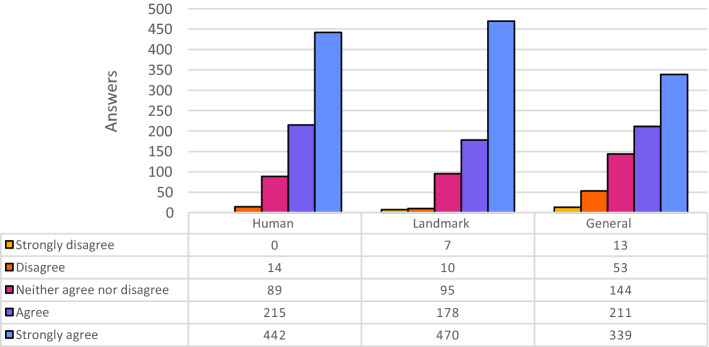
Evaluation of Wikipedia descriptions, partitioned for category of images

Figure 2 shows the distributions of the answers provided by the participants while evaluating the quality of Wikipedia alt-texts. Each distribution summarizes 760 answers since the 30 images were split into three categories, with 10 images per category. The Wikipedia alt-texts pertaining to images representing Human beings and Landmark scenarios obtained very high evaluations. In these two cases, Wikipedia alternative texts have more than half of the total answers marked as “Strongly Agree”. Also for the General category the results are very good, although slightly less positive.

### Azure Computer Vision Engine

**Fig. 3:**
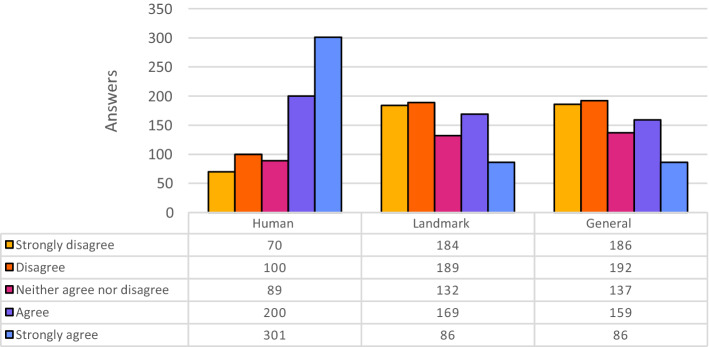
Evaluation of Azure CVE descriptions, partitioned for category of images

Figure 3 shows the distributions of the answers provided for Azure CVE. This AI engine gets good results for pictures of Human beings. Indeed, facial recognition is one of the most powerful features provided by this tool. Almost all famous people of the 19th and 20th century included in the experiment were successfully recognized. For example, the picture of John Lennon^29^ was labeled as “John Lennon in glasses looking at the camera” (see Table 9).

**Table 9:** Captions for the picture of John Lennon

Wikipedia	John Lennon 1969
Azure CVE	John Lennon in glasses looking at the camera
Amazon Rek	face, person, accessories, glasses, beard
Cloudsight	man in black framed eyeglasses
Auto Alt-Text	a man with a tie

For the other two categories, the histograms show worse performances. In the Landmark category, there is a relevant number of answers “Mostly disagree” and “Disagree” as well as for the General category.

### Amazon Rekognition

**Fig. 4:**
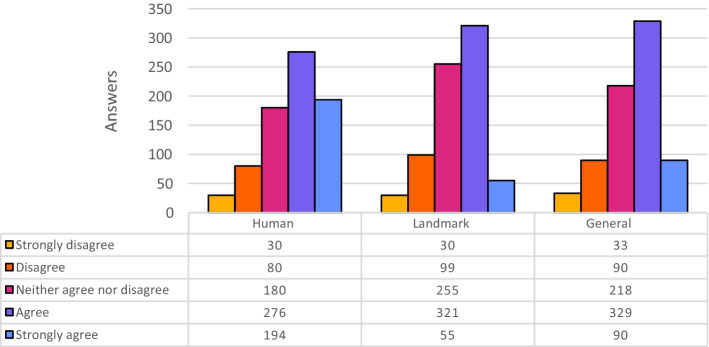
Evaluation of Amazon Rek descriptions, partitioned for category of images

Figure 4 shows the distributions of the answers for Amazon Rek. The results returned by this online service are different, because they are composed by sequences of tags.

Using different types of alt-text representations is another aspect behind this experiment. From the results (see Fig. 4), it is evident that most of the answers are centered around good values of the scale. In particular, “Agree” is the most voted option followed by “Neither agree nor disagree”. This could be explained by the fact that the majority appreciated the quality of this tag-based representation, even though previous research by Enge [6] suggests that they would have not used the same tags to describe the images.

### Cloudsight

**Fig. 5:**
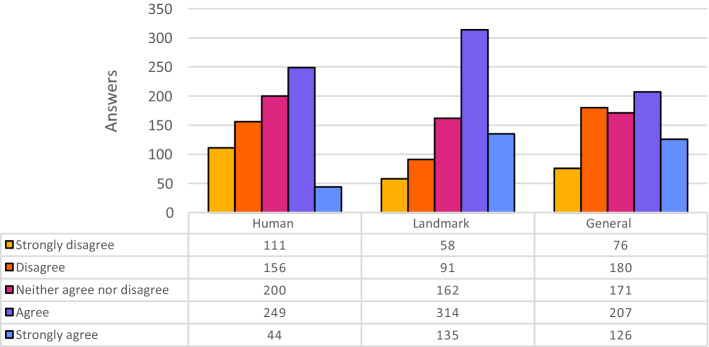
Evaluation of Cloudsight descriptions, partitioned for category of images

Figure 5 shows the distributions of the answers for Cloudsight.

This tool could not recognize famous people, but the descriptions were almost always faithful to reality. For example, the result caption for the picture of John Lennon is “man in black framed eyeglasses”. Students have rated these alternative texts in different ways, sometimes with good grades (Fig. 5).

With this tool, the best results are obtained by the images in the Landmark category. Most of the time, the algorithm could not recognize important cities or famous places, but it provided acceptable descriptions with many details.

For example, for the main Wikipedia figure representing the Palacio de Bellas Artes^30^, a prominent cultural centre in Mexico City, the description generated by Cloudsight is “people walking near beige concrete building under blue sky during daytime”, but the tool could not recognize the palace. All the captions for such image are reported in Table 10.

**Table 10:** Captions for the image of the Palacio de Bellas Artes, Mexico City

Wikipedia	Bellas Artes
Azure CVE	a large building with Palacio de Bellas Artes in the background
Amazon Rek	building, architecture, person, mansion, housing, house, palace
Cloudsight	people walking near beige concrete building under blue sky during daytime
Auto Alt-Text	a tall building with a clock on it

Concerning the General category, sometimes the descriptions are good and appreciated but often they are also perceived as not accurate or totally wrong. These outcomes were unexpected because the purpose of Cloudsight is object recognition, and therefore, we expected good performance in this generalist category.

### Auto Alt-Text for Google Chrome

**Fig. 6:**
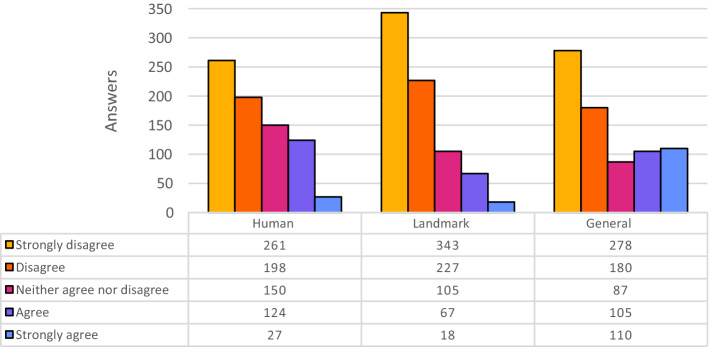
Evaluation of Auto Alt-Text descriptions, partitioned for category of images

Finally, Fig. 6 shows the distributions of the answers provided by the participants while evaluating the quality of the descriptions generated by Auto Alt-Text.

This is the tool that scored worst: its outputs are confusing and inaccurate, often objects or people in the images are not correctly recognized. The results of the experiment clearly show this fact, highlighting the “strongly disagree” value as the most popular (see the left-most bars in the histograms in Fig. 6).

Only for some General pictures, there were acceptable outcomes, like the description generated for the main Wikipedia figure for the Sports team entry^31^, i.e., “a group of young men playing a game of soccer”. All the captions for such image are reported in Table 11.

**Table 11:** Captions for the image of children playing soccer

Wikipedia	youth soccer indiana
Azure CVE	a group of young men playing a game of football
Amazon Rek	person, people, team, soccer, ball, sport, team sport, soccer ball, football
Cloudsight	three boys playing soccer on green grass field during daytime
Auto Alt-Text	a group of young children playing a game of soccer

### Answers to the research questions

The analyses performed to answer the two Research Questions introduced in Sect. 1 are discussed in the following sections. Since we observed that the quality of the descriptions varies for the three categories of images, we decided to perform each analysis separately.

#### Human category

Table 12 reports the key statistics for Human subjects. Azure CVE is the best tool, Amazon Rekognition ranks second with -0.05 points. This means that both tools are able to generate descriptions for images with human beings with a high perceived quality. Cloudsight gets the third position but with a reduction of -0.79. Finally, Auto Alt-Text is the worst among the four tools even if it achieves a slightly better score with respect to to its global mean (+0.06, see Table 5).

**Table 12:** Human category: statistics

	Wikipedia	Azure	Amazon	Cloudsight	Auto
		CVE	Rek		Alt-Text
Mean	4.43	3.74	3.69	2.95	2.29
Median	5.00	4.00	4.00	3.00	2.00
StDev	0.77	1.34	1.08	1.16	1.20

By comparing these results with the perceived correctness of the ground truth descriptions from Wikipedia, it is evident that numbers are heavily favoring the latter. Indeed, Wikipedia scored + 0.69 points with respect to Azure CVE and nearly reaches the double of the worst tool. It is also worth noting that the median value of the answers of Wikipedia is 5 out of 5.

**Fig. 7:**
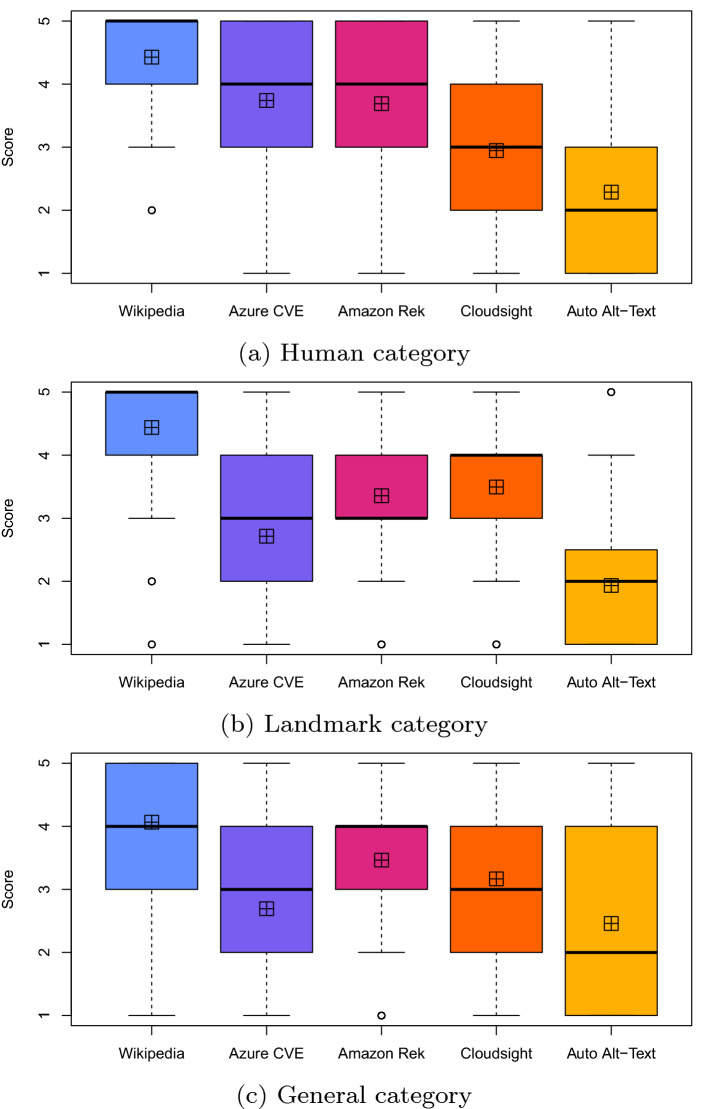
Boxplots summarizing the distributions of the answers provided by the study participants. The straight black line represents the median value while the crossed square represents the average value. Each answer has a value in a 5-point Likert scale. The colors for the various boxplots are chosen from the IBM design library “color blind safe” color palette [10]

A more detailed view of the distributions can be seen in the boxplots shown in Fig. 7a. From them, it is evident that the results for Azure CVE and Amazon Rek are almost identical while the other two tools obtained different and lower scores. The boxplot of Wikipedia alt-texts clearly shows a very high evaluation.

Table 13 reports the Wilcoxon test used to compare the effects of the treatments (i.e., the tools and Wikipedia) on each subject with a paired analysis where, for each image, each possible pair of descriptions is compared. From the table, it is evident that for all the pairs of treatments, the *p*-value is negligible (i.e., < 0.01) except for the pair $\langle$Azure CVE, Amazon Rek $\rangle$ that achieved a *p*-value of 0.27. Thus, excluding this specific case, the differences in terms of perceived correctness of the descriptions are statistically significant in all the other cases. Therefore, we can reject the null hypotheses $H_{0}$ and accept $H_{a}$ for every pair of treatments except for $\langle$Azure CVE, Amazon Rek $\rangle$. This means that, with the exception of one single case, the participants assigned a correctness score to the various descriptions that are statistically different with respect to the considered tool.

Concerning Wikipedia, the difference is always statistically significant, as expected, given the evident difference in the distribution of the answers.

**Table 13:**
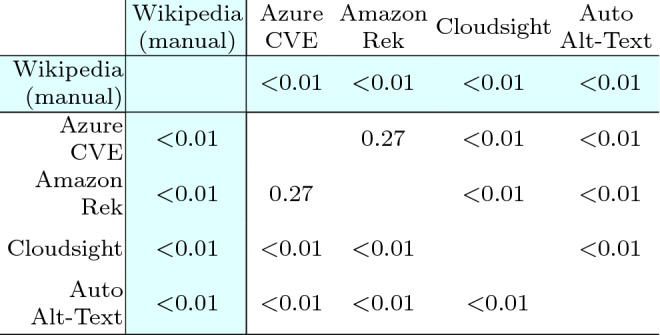
Human category: Wilcoxon test statistics, each cell reports the *p*-value computed by using the Wilcoxon paired test among the two corresponding distributions of answers

The difference between two distributions is considered statistically significant when the *p*-value is $< 0.05$. The cells with white background contain data to answer RQ1, those with colored background pertain to RQ2

To better analyze the magnitude of the significant differences determined with the Wilcoxon statistics, we report in Table 14 the Cliff’s delta effect size computed for all pairs of treatments. We can observe that for all pairs where the difference is statistically different, |*d*| assumes values from 0.31 to 0.59 (i.e., from small to large). Such values correspond to the smallest difference, i.e., the case of the pair $\langle$Cloudsight, Auto Alt-Text $\rangle$, and the largest one, i.e., the case of the pair $\langle$Amazon Rek, Auto Alt-Text $\rangle$. The magnitude of such differences is clearly visible also by observing Fig. 7a. Concerning Wikipedia, the magnitude of the differences varies from 0.27 to a very high 0.82, and the results confirm what can be observed by analyzing the distributions shown in the boxplots of Fig. 7a.

**Table 14:**
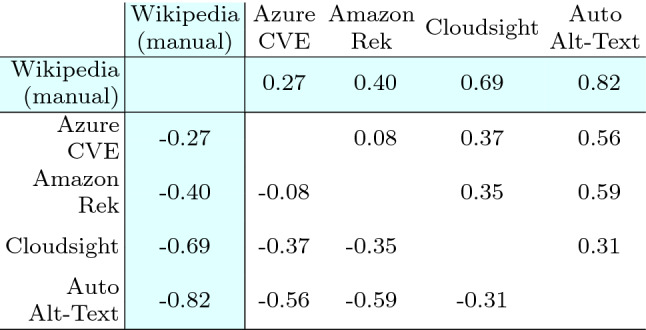
Human category: Cliff’s delta statistics, the effect size is considered small for $0.148 \le |d| < 0.33$, medium for $0.33 \le |d| < 0.474$ and large for $|d| \ge 0.474$, see [8]

The table is symmetric with respect to the diagonal except for the sign of the values that simply depend on the order of the treatments (the sign says which treatment dominates the other i.e., whether X is prevailing on Y or vice-versa). The cells with white background contain data to answer RQ1, those with colored background pertain to RQ2

*RQ1 (Human)* To summarize the results achieved by the four tools on the Human category, we can observe that two of them—Azure CVE and Amazon Rek—performed similarly and scored the top evaluation concerning the correctness of the descriptions they generate. Cloudsight is in the third position with a mean score of about -0.8 points (out of 5) with respect to the top two. Finally, Auto Alt-Text obtained the lowest evaluation with a mean score lower of about -1.4 points. All differences are statistically significant except for the pair $\langle$Azure CVE, Amazon Rek $\rangle$ that obtained very similar results (with a difference of -0.05 points only).

*RQ2 (Human)* To summarize the results achieved by Wikipedia on the Human category, numbers show that there is always a statistically significant difference in the perceived correctness between the ground truth descriptions provided by the online encyclopedia and those provided by the various tools. Although there is a big difference among the tools (as seen for RQ1), even the top two are quite outdistanced from Wikipedia.

#### Landmark category

Table 15 reports the key statistics for the Landmark category. In this case, Cloudsight is the best tool, closely followed by Amazon Rek (-0.14). Azure CVE occupies the third position (-0.78), and Auto Alt-Text is outdistanced with a score close to the half (-1.57). Cloudsight is able to generate descriptions for Landmark images with a perceived quality which is higher than for the other categories.

By comparing the results with the perceived correctness of the human-authored descriptions, it is again evident that the numbers are heavily in favor of Wikipedia. Indeed, the Wikipedia alt-texts scored +0.94 points with respect to the best tool and more than double the score obtained by the worst tool. Also in this case, the median value of the answers for Wikipedia is 5.

**Table 15:** Landmark category: statistics

	Wikipedia	Azure	Amazon	Cloudsight	Auto
		CVE	Rek		Alt-Text
Mean	4.44	2.72	3.36	3.50	1.93
Median	5.00	3.00	3.00	4.00	2.00
StDev	0.83	1.35	0.93	1.14	1.07

A more detailed view on the distributions can be seen in the boxplots of Fig. 7b, showing similar results for Cloudsight and Amazon Rek. Azure CVE has a strong variability, with some descriptions scoring very good results and others by far lower, and Auto Alt-Text consistently achieves the lowest results. Also for this category, the boxplot of Wikipedia shows the very high evaluations obtained by its alt-texts.

Table 16 reports the results of the Wilcoxon test: for all pairs of treatments the *p*-value is negligible (i.e., <0.01). Thus the differences in terms of perceived correctness of the captions are statistically significant in all the cases and we can reject the null hypotheses $H_{0}$ and accept $H_{a}$ for every pair of treatments. The respondents assigned a correctness score that is statistically different with respect to the considered tool. Concerning Wikipedia alt-texts, the difference is always statistically significant.

**Table 16:**
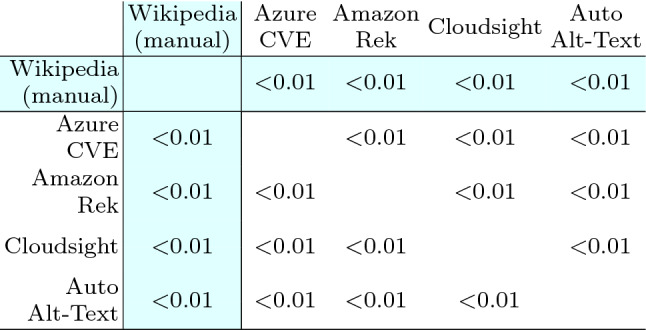
Landmark category: Wilcoxon test statistics

As before, we report in Table 17 the Cliff’s delta effect size. For all pairs of treatments |*d*| assumes values from 0.11 to 0.65 (i.e., from small to large). Such values correspond to the smallest difference, i.e., the case of the pair $\langle$Cloudsight, Amazon Rek $\rangle$, and the largest ones, for $\langle$Auto Alt-Text, Amazon Rek $\rangle$ and $\langle$Auto Alt-Text, Cloudsight $\rangle$. The magnitude of such differences is also visible by observing Fig. 7b.

With the exception of Cloudsight, for Wikipedia the magnitude of the differences is higher for Landmark images compared to Human. Indeed |*d*| varies from 0.50 (already above the large threshold) to a very high 0.88. Also in this case, the results confirm what can be observed by analyzing the distributions in the boxplots of Fig. 7b.

**Table 17:**
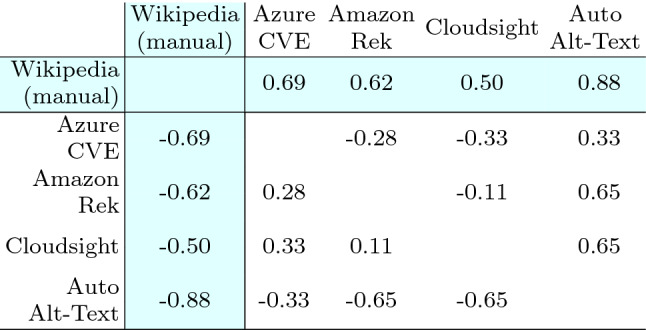
Landmark category: Cliff’s delta statistics

*RQ1 (Landmark)* To summarize the results achieved by the four tools on the Landmark category, we can observe that two tools—Cloudsight and Amazon Rek—performed very similarly and scored the top evaluation concerning the correctness of the descriptions they generate. Azure CVE is in the third position with a mean score lower of about − 0.8 points with respect to the top two. Finally, Auto Alt-Text got the lowest evaluation with a mean score lower of about − 1.6 points. All the differences are statistically significant.

*RQ2 (Landmark)* To summarize the results achieved by Wikipedia on the Landmark category, numbers show that there is always a statistically significant difference in the perceived quality between the ground truth descriptions provided in the online encyclopedia and those provided by the various tools. Although there is a big difference among the tools (as seen for RQ1), even the top two are quite outdistanced from Wikipedia.

#### General category

Table 18 reports the key statistics for the General category. Amazon Rek is the best tool, closely followed by Cloudsight (− 0.29). Azure CVE is in third position (− 0.77), and Auto Alt-Text is outdistanced in fourth position (− 1.00).

**Table 18:** General category: statistics

	Wikipedia	Azure	Amazon	Cloudsight	Auto
		CVE	Rek		Alt-Text
Mean	4.07	2.69	3.46	3.17	2.46
Median	4.00	3.00	4.00	3.00	2.00
StDev	1.03	1.34	0.99	1.24	1.46

By comparing these results with those of Wikipedia, it is evident that the latter is still the winner even if the difference is reduced. For this category, the human-authored alt-texts scored +0.61 points with respect to the best tool, Amazon Rek, and outdistanced (+1.61) the score obtained by the worst tool, Auto Alt-Text. However, in this case, the median value of the answers is 4 (like Amazon Rek).

The boxplots in Fig. 7c show that Amazon Rek performed best among the tools. Cloudsight and Azure CVE have similar distributions, but with different mean values (see the crossed squares in the figure). Auto Alt-Text consistently scored worst, but with a quite sparse distribution (StDev = 1.46): in some cases, the correctness of the descriptions was not perceived so bad. Concerning Wikipedia, the boxplot clearly shows high evaluations but this time the distribution is more sparse: respondents rated the correctness of the alt-texts as lower for several images.

Table 19 reports the last Wilcoxon test used to compare the effects of the treatments on each subject. Again, all the *p*-values are <0.01 and, therefore, the differences in terms of perceived correctness of the generated captions are statistically significant, for all tools and Wikipedia, and we can reject the null hypotheses $H_{0}$ and accept $H_{a}$ for each pair of treatments.

**Table 19:**
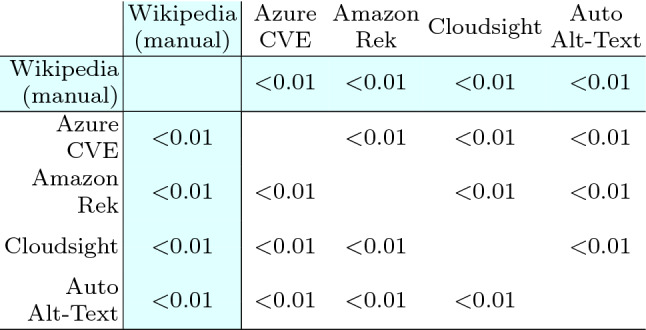
General category: Wilcoxon test statistics

The Cliff’s delta effect size is shown in Table 19. For all pairs |*d*| assumes values from 0.11 to 0.41 (i.e., from small to medium) which correspond, respectively, to the smallest difference, i.e., the case of the pairs $\langle$Azure CVE, Amazon Rek $\rangle$, and $\langle$Azure CVE, Cloudsight $\rangle$, and the largest one, i.e., the case of the pair $\langle$Amazon Rek, Auto Alt-Text $\rangle$ (see also Fig. 7c).

For Wikipedia, the magnitude of the differences is slightly lower for this category and |*d*| varies from 0.34 (just above the medium threshold) to 0.59 (high), confirming what can be observed in the boxplots of Fig. 7c where the distribution of Wikipedia is in general more overlapping (Table 20).

**Table 20:**
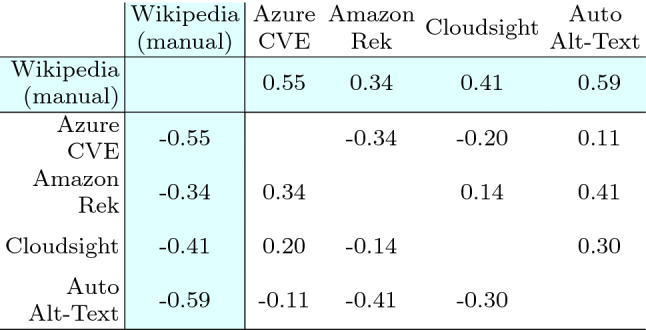
General category: Cliff’s delta statistics

*RQ1 (General)* For the General category of images, we can observe that Amazon Rek reached the best performance followed by Cloudsight. Then, Azure CVE is in the third position with a mean score of about − 0.8 points with respect to the top one. Finally, Auto Alt-Text obtained the lowest evaluation, but the absolute value of the score is not far from that of Azure CVE, and exactly − 1 point from the best tool. All the differences are statistically significant.

*RQ2 (General)* To summarize the results achieved by Wikipedia on the General category, numbers show that there is always a statistically significant difference in the perceived quality between the ground truth descriptions provided by the online encyclopedia and those provided by the other tools. In this particular category of images, even if Wikipedia alt-texts are perceived as the most correct, the distance with respect to the best tool is not so big. In some cases, the evaluations are very similar. This is evident since the distribution of Wikipedia has a not negligible overlap with the others, in particular with that of Amazon Rek.

### Discussion

From the analyses of the previous sections, it is clear that the descriptions generated by the various tools have a lower perceived correctness than those written by Wikipedia contributors. Among the tools, some differences could be observed. Amazon Rek has the highest perceived correctness followed by Cloudsight and Azure CVE. The last position is always occupied by the Chrome extension Auto Alt-Text. It is also interesting to note that the correctness of the automatic captions varies depending on the category of the images. Table 21 summarizes the ranking of the various categories.

**Table 21:** Summary of the comparison: ranking of Wikipedia (always the best) and the four tools

	Wikipedia	Azure	Amazon	Cloudsight	Auto
		CVE	Rek		Alt-Text
Human	1	**2**	3	4	5
Landmark	1	4	3	**2**	5
General	1	4	**2**	3	5
Overall	1	4	2	3	5

We found that some tools show good performance on a particular category (see number 2 in bold in Table 21), but do not perform as well on others (e.g., Azure CVE on images in the categories Landmark and General). On the contrary, other tools perform consistently quite well (Amazon Rek) or quite badly (Auto Alt-Text). This behavior probably depends on the characteristics of the AI algorithms used to produce the descriptions and on the data sets used for training them. Some solutions can be particularly specialized in recognizing people with a high level of precision and, at the same time, they do not perform as well on other categories of subjects.

The differences observed in the perceived correctness of the descriptions can also depend from the process employed by the tools. Indeed, some of them can be used for generating descriptions in *batch* mode, for instance, when a web page is about to be published. In such case, the tool could employ more sophisticated solutions since the execution time is not relevant (e.g., obtaining a description in a few minutes is acceptable). On the other hand, browser add-ons like Auto Alt-Text, designed to support the web navigation of visually impaired users by generating captions *on-the-fly*, must be very fast. This requirement can partially balance a lower quality of the results.

Given our findings, we believe that the producers of web content should, as the first choice, add manually image descriptions as much as possible. Our study clearly shows that, with the current technology, the correctness (and thus the perceived quality) of the human-authored descriptions is simply not yet reachable by automated tools. As a second choice, state of the practice solutions allow to produce image descriptions of good quality: in such cases, web content producers should investigate and experiment which of them perform better on specific types of images they have to publish since, as highlighted, the quality of the descriptions can vary depending on the image types. Finally, following the best practices, we do not recommend leaving the alt-text empty, also because the on-the-fly solution we evaluated provided the worst results.

Moreover, besides not being effective from a quality point of view, on-the-fly tools are not efficient from an energy consumption point of view. Popular pages containing images without alt-texts would require calling remote APIs for generating their captions for a large number of times. On the contrary, this task could be done only once, server-side, during the first publication of new web pages, achieving more sustainable websites.

### Threats to validity

This section discusses the threats to validity that could affect our results: *internal*, * conclusion* and *external validity*.

*Internal validity* threats concern factors that may affect a dependent variable (in our case, *Correctness*). To avoid any bias in the evaluation of the various descriptions, they were presented in random positions. In this way, participants could not associate them to a particular tool since they could not consider their repetitive orderings. Moreover, since the participants had to evaluate five descriptions for each of the 30 images, a fatigue effect may intervene. However, since for each image we required the participants to evaluate all the five descriptions, we can exclude a fatigue effect on the results (it cannot affect the descriptions of the various tools differently). Another threat concerns the English skills of the participants that, even if they can be judged as very good, are not at the level of a mother tongue (see Sect. 4.3). To reduce this threat, we suggested participants, if necessary, to use a dictionary/translator for the descriptions to avoid language troubles such as a word in the description whose meaning was not clear/known (e.g., a peculiar word describing a specific object). Note that, in our opinion, the simple descriptions generated by the tools make the participants’ skill more than adequate for the evaluation. Finally, three tools return sentences while only one (Amazon Rek) returns a list of tags. We asked participants to evaluate each description with respect to a picture considering aspects like the correctness and the precision of the description. We do not think that having a list of tags or a complete sentence can change, per se, the evaluation provided by the participants. However, since we have not two versions of the same descriptions (tag-based vs. sentence-based) to compare with an experiment, we cannot be sure of that.

Threats to *conclusion validity* concern issues that may affect the ability of drawing a correct conclusion. They can be due to the sample size of the experiment (76 participants, 2280 evaluations) that may limit the capability of statistical tests to reveal any effect and to the chosen statistical tests themselves. In this experiment, we decided to use nonparametric tests for testing the effect of the main factor due to the size of the sample and because we could not safely assume normal distributions [15].

Threats to *external validity* can be related to: (i) the choice of the images and (ii) the use of students as experimental participants. For the choice of the images, we devised the procedure described in Sect. 4.1, to avoid any bias in the choice and guarantee transparency in the experiment. The three categories of images, although they do not cover all types of images, are certainly of great interest as they represent a significant fraction of images that can be found on standard websites. As far as the participants are concerned, being students in computer science, they have a good knowledge of the English language (they all have a course of English language in their study plan and are used to reading technical documentation in English). For this reason, even if they cannot have the same level of comprehension as a native speaker, on simple descriptions composed by just a few words, their comprehension level is probably not so different (we also suggested them to access the web in case they needed clarifications on the meaning of specific words).

## Conclusion and future work

The Alternative text is one of the most effective ways to help visually impaired people to understand what a picture on a web page represents. However, web developers often underestimate this critical information, making their websites not equally accessible to all users.

During the last few years, thanks to the advent of advanced machine learning techniques, scientific research has made significant steps forward in this field by proposing different approaches to automatic image captioning. At the same time, companies like Microsoft and Amazon have developed services, usually available through APIs, that are able to process images and return textual descriptions. However, to the best of our knowledge, these tools were never evaluated to understand if their outputs could be used as a substitute for manual defined alt-texts in the web context. For this reason, the goal of our experiment was to evaluate the perceived correctness of the generated descriptions by comparing them with human-defined references.

Specifically, we selected four well-known tools having heterogeneous characteristics: Azure Computer Vision Engine, Amazon Rekognition, Cloudsight, and Auto Alt-Text for Google Chrome. Using 60 images taken from Wikipedia, we asked 76 survey participants to evaluate their descriptions without knowing the source.

The overall outcomes show that, on average, people still prefer human-authored texts even when the tools’ descriptions are accurate. Indeed, the generated descriptions have not yet reached a level of precision comparable to those written by humans. Experts of the field can find the obtained results quite predictable. However, a valuable contribution of the present study is that it provides an estimate of the magnitude of such differences with a detailed analysis from multiple perspectives. Indeed, we analyzed different categories of images (Human, Landmark and General) and their descriptions generated by different tools. In this way, it was possible to evaluate how the quality of the descriptions varies depending on multiple factors. The analysis also highlighted the unreliability of some algorithms: they generate descriptions that sometimes are not enough precise or even wrong. Among the tools examined, an algorithm which is mature enough to replace the manual writing of alternative texts does not exist. However, some algorithms have shown their ability to generate good descriptions for specific categories of images. Therefore, they could be used as support tools to the usual manual work of web developers.

Possible future extensions for this study include: (1) considering the descriptions generated by other tools available on the market; (2) extending the number of participants to get a more precise judge about the quality of their outputs; (3) considering different languages in order to understand if the results differ depending on the localization of websites; (4) it would also be extremely interesting to define a procedure for including visually impaired users in the evaluation process. Moreover, we hope that other experts can use our work as a starting point for more detailed analysis considering for instance: (1) the technical reasons influencing the results provided by the considered tool (i.e., machine learning aspects), and (2) the linguistics aspects influencing the results (e.g., with the same semantic content, which kind of description is perceived more effective to describe an image and why).

Finally, we described in this paper an experiment done on images belonging to generic categories. As a future work, we plan to organize a second experiment to investigate what happens in the case of STEM images. These are generally complex images, showing for example graphs, charts, diagrams, maps, etc., which contain substantial information that cannot be conveyed in a short phrase or a sequence of tags. Labeling these images is a complex task even for the humans. However, as educators, we must guarantee that visually impaired students have access to the same content of their sighted peers, and therefore we find extremely useful to perform research in this direction.

## Footnotes

1 https://www.statista.com/statistics/278414/number-of-worldwide-social-network-users/

2 WAI, https://www.w3.org/WAI/

3 According to the MDN Web Docs website longdesc is no longer recommended, see https://developer.mozilla.org/en-US/docs/Web/API/HTMLImageElement/longDesc.

4 https://www.mturk.com/

5 https://vizwiz.org/

6 https://achecker.achecks.ca/checker/index.php

7 science, technology, engineering and mathematics

8 #A11y is another way of writing the word accessibility. It is an abbreviated version for Twitter and other social network sites that limit the number of characters.

9 https://cloud.google.com/vision/

10 https://www.ibm.com/watson/

11 The top right image in https://en.wikipedia.org/wiki/Sugar

12 https://azure.microsoft.com/en-us/

13 https://azure.microsoft.com/en-us/services/cognitive-services/computer-vision/

14 https://docs.microsoft.com/en-us/azure/cognitive-services/

15 https://blogs.microsoft.com/ai/azure-image-captioning/

16 https://azure.microsoft.com/en-us/services/cognitive-services/computer-vision/#features

17 https://aws.amazon.com/rekognition/

18 https://aws.amazon.com/machine-learning/ai-services/

19 https://cloudinary.com/blog/making_media_accessible_how_to_automatically_generate_alt_text_for_images

20 https://cloudsight.ai/

21 https://chrome.google.com/webstore/detail/auto-alt-text/lgmkhmedpaidhgonghfhijjlgaballje

22 https://en.wikipedia.org/wiki/Milk

23 https://en.wikipedia.org/wiki/Wikipedia:Vital_articles/

24 https://en.wikipedia.org/wiki/Forbes_Celebrity_100

25 We slightly adapted such descriptions by removing any reference to the file type (e.g., the fact that the image is in jpg or png format), and fully translating them in English in case some words were reported in a different language (obviously excluding from any translation proper names of people and landmarks)

26 https://moodle.org/

27 https://en.wikipedia.org/wiki/Common_European_Framework_of_Reference_for_Languages

28 The top right image in https://en.wikipedia.org/wiki/New_York_City

29 The top right image in https://en.wikipedia.org/wiki/John_Lennon

30 The top right image in https://en.wikipedia.org/wiki/Palacio_de_Bellas_Artes

31 The top right image in https://en.wikipedia.org/wiki/Sports_team
